# General practice patients starting treatment for substance use problems: observations from two data sources across levels of care

**DOI:** 10.1186/s12889-020-09038-0

**Published:** 2020-06-18

**Authors:** Nicole Boffin, Jerome Antoine, Luk Van Baelen, Sarah Moreels, Kris Doggen

**Affiliations:** Department of Epidemiology and Public Health, Sciensano, Brussels, Belgium

**Keywords:** Substance-related disorders, Public health surveillance, Patient care

## Abstract

**Background:**

In Belgium, the incidence of treatment episodes for substance use problems is monitored by the Network of Sentinel General Practices (SGP), and at higher, specialist care levels by the Treatment Demand Indicator (TDI) surveillance. Using both data sources, we examine 1) how patients starting specialist treatment for substance use problems on referral by their GP compare to those that were referred by non-GP caregivers; 2) how patients starting GP treatment for substance use problems without receiving concurrent specialist treatment compare to those who did.

**Methods:**

Both surveillances are based on the TDI protocol for reporting data to the European Monitoring Centre for Drugs and Drug Addiction (EMCDDA) on individuals starting treatment as a result of their substance use. Data from 2016 and 2017 were examined using 95% confidence intervals and multivariate logistic regression.

**Results:**

According to TDI-data (*n* = 16,543), determinants of being referred by a GP (versus by a non-GP caregiver) for specialist treatment were age ≥ median (OR 1.25; 95% CI 1.13–1.38), education ≥ secondary level (OR 1.27; 95% CI 1.15–1.41), recent employment (OR 1.71; 1.56–1.88), recent stable accommodation (3.62; 95% CI 3.08–4.26), first treatment episode (OR 1.72; 95% CI 1.57–1.87), recent daily primary substance use (OR 1.46; 95% CI 1.33–1.59) and mono substance use (OR 1.23; 95% CI 1.04–1.48). Type of substance use was a significant determinant with higher odds of using pharmaceuticals (and alcohol) (OR 1.24; 95% CI 1.04–1.48), and lower odds of using cannabis only/primarily (OR 0.73; 95% CI 0.62–0.86), with reference to street drugs minus cannabis only/primarily. According to SGP data (*n* = 314), determinants of starting GP treatment without concurrent specialist treatment were recent employment (OR 2.58; 95% CI 1.36–4.91), first treatment episode (OR 2.78; 95% CI 1.39–5.55) and living in the Brussels or Walloon region (OR 1.97; 95% CI 1.06–3.66).

**Conclusions:**

This study provides a useful insight into the general practice population treated for substance use problems. It shows that both surveillances consistently found a relatively favourable profile of general practice patients with substance use problems.

## Background

In many European countries the epidemiology of substance use problems is described using data about the population that started treatment for those problems. The main reason is that traditional population surveys are less reliable as far as the use of street drugs and alcohol is concerned. The Treatment Demand Indicator (TDI) was implemented on behalf of the European Monitoring Centre for Drugs and Drug Addiction (EMCDDA) to collect data in a standardized way in the European Union, Turkey and Norway [1].

Belgium started collecting TDI data from 2011 on, including also data from patients with alcohol problems only or primarily [2]. The Belgian TDI covers specialist treatment for substance use problems, excluding GP treatment because of feasibility issues. It is well-established that GPs do provide care to patients with substance use problems. In 2001–2 the European Study of the Epidemiology of Mental Disorders (ESEMeD) found that 17% of the Belgian population with an alcohol disorder had been receiving professional care [3]. GPs were consulted most often: 66% of people seeking care for an alcohol disorder consulted a GP and 10% consulted only a GP [3]. GPs play a key role in Belgian healthcare, even though patients are basically free to consult a care provider of their choice. Overall, 95% of the general population in Belgium has a regular GP [4].In 2016, following a successful pilot study, the surveillance of new treatment episodes for substance use problems was taken up by the Network of Sentinel General Practices (SGP), using an adapted TDI protocol [5]. Having acquired data from both surveillance systems over a period of 2 years (2016–2017), we decided to examine to what extent general practice patients data from both sources are in agreement and whether differences are plausible or consistent with the body of evidence, e.g. health problems presented in primary care are less severe/complex than those at higher, specialist care levels [6]. Comparing data sources is one way to explore the quality of data and may yield additional information for health policy. Our assumption is that the population starting GP treatment for substance use disorders shares core characteristics with the population referred by a GP for specialist treatment. In line with that reasoning, we assumed that general practice patients receiving mixed treatment share more traits with patients starting specialist treatment than they do with patients starting GP treatment without receiving concurrent specialist treatment. To minimize population heterogeneity, we limited the TDI study population to individuals who were referred by caregivers to start specialist treatment, in other words, individuals who had already sought professional care for their substance use problems.Using unpooled data from the SGP and TDI surveillances, this study examines 1) how patients starting specialist treatment for substance use problems on referral by their GP compare to similar patients referred by non-GP caregivers; and 2) how patients starting GP treatment for substance use problems without receiving concurrent specialist treatment compare to those who did.

## Methods

Key methodological features of the TDI and SGP surveillance studies are summarised in Table 1.

**Table 1:** Key methodological features of the TDI and SGP surveillance studies

	SGP	TDI
**Setting**	General practice (primary care)	Specialist care for substance use problems (secondary and tertiairy care)
**Study design**	Sentinel surveillance by a sample of general practices	Register completed by caregivers from participating centres
**Data collection tools**	Standard patient data form/questionnaire to complete by caregivers for every patient starting a new treatment episode	
**Data transfer**	Postal mail, data entry by Sciensano	Online standard forms to transfer data record by record, or, repository tool to transfer patient data files for a given year

### Settings and study participants

The TDI and the SGP surveillance systems of provider-reported, care-based data are managed within Sciensano, the Belgian Institute for Health.

The Belgian Network of SGP consists of a sample of GPs who report the occurrence and characteristics of well-defined health-related events in their daily practice [7]. Data are reported weekly on standard forms for a period of at least 1 year. Across the study period, the network comprised 125 practices. Annual statistics showed that sentinel GPs are comparable to non-sentinel GPs for age and gender. The network covers about 1,5% of the Belgian population in most Belgian districts [8]. As Belgian GPs do not serve a defined practice population, the size of the SGP patient population - the denominator - is estimated by applying the ratio of patient contacts across the entire Belgian population to the sum of weekly patient contacts in the network.The Belgian TDI study protocol has recently been described in detail [2]. The TDI register collects socio-demographic, treatment- and substance-related data about patients who started treatment for substance use disorders in a wide range of settings. Patients are interviewed by health professionals by means of a structured questionnaire. In order to detect multiple treatment episodes in the same patients, national identification numbers are used in accordance with the European General Data Protection Regulation. Data are gathered by Sciensano using a reporting module or a repository tool allowing batch transfer of data.

Participation in the TDI surveillance is only mandatory for particular types of treatment centres for substance use problems. Throughout the study period, 221 treatment centres reported cases. TDI data from 2014 show that the best participation rates were reached by centres that are specialised in substance use problems (56 to 100% of eligible centres) [2]. Participation rates were lower among hospitals and centres that also offer treatment for other mental health problems (17 to 52% of eligible centres).

In contrast to the SGP surveillance, the TDI surveillance is unable to estimate the incidence of treatment episodes for substance use problems in the total population for lack of data about the size of the denominator, that is the population covered by the register.

### Patient population samples

To address our first research question, we selected data about the TDI patients that were referred or encouraged to seek specialist treatment by caregivers. To address our second research question, we included data from all the SGP patients.

### Case definitions

The SGP instructions were based on the Belgian TDI protocol [2]. A new treatment episode starts with the first face-to-face contact with a GP/other caregiver for substance use problems. When the patient shows up with a similar treatment demand 6 months after the previous face-to-face contact, a new treatment episode starts. Treatment was defined as any activity directly targeting patients with substance use problems in order to ameliorate their mental, medical or social status. We explicitly described possible GP interventions aimed at reducing substance-related harm in active users, detoxification or abstinence, medical and non-medical problems, informal advice, counselling and support (e.g. a brief intervention). Excluded were interventions only targeting the physical consequences of substance use (e.g. infections or overdoses) or focusing mainly on problems other than substance use.

### Variables

The variables in this paper (all described in Table 2) are (derived from) the items 1–6, 9–12, 14–15 and 17 of the TDI protocol 3.0 [1]. In the context of employment, accommodation and use of primary/only substance, ‘recent’ was understood as the last 30 days before the start of the new treatment episode. Patients who had recently been living at different places (friends’ home, street, shelters, etc.) or moved from one place to another, were considered as residing in unstable accommodation. Four variables were not recorded by the SGP, respectively treatment centre type, source of referral, highest educational level completed and recent accommodation. The variable ‘primary drug’ was reported in less detail by the SGP, e.g. the groups ‘cocaine or crack’ and ‘cannabis’ comprise three subcategories in the TDI. One additional variable was reported by the SGP, i.e. whether or not the patient was concurrently receiving specialist treatment for substance use problems.

**Table 2:** Characteristics of new treatment episodes of substance use problems by data source: the TDI subpopulation referred/motivated by caregivers and the SGP population, Belgium 2016–7

	TDI subpopulation of patients referred by caregivers (*N* = 16,543)		SGP population (*N* = 314)	
	n/valid N	%	n/valid N	%
Sex				
Man	11,476/16,543	69.5	220/314	70.1
Age				
< 20	674/16,543	4.1	14/312	4.5
20–29	2982/16,543	18.1	35/312	11.2
30–39	4518/16,543	27.4	67/312	21.5
40+	8327/16,543	50.5	196/312	62.8
Highest educational level completed				
None or primary	3865/13,877	27.9		
Secondary	7519/13,877	54.2		
Tertiary	2493/13,877	18.0		
Recent stable accommodation	13,075/16,219	80.6		
Recently employed	3394/14,989	22.6	135/292	46.2
Region				
Flanders	9936/16,543	60.0	198/314	63.1
Wallonia	4495/16,543	27.1	82/314	26.1
Brussels	2112/16,543	12.8	34/314	10.8
Previous treatment	10,952/16,039	68.3	178/282	63.1
Type of substance use				
Alcohol only (I)	7354/16,543	44.5	176/314	56.1
Pharmaceuticals (and alcohol) (II)	1047/16,543	6.3	46/314	14.7
Cannabis only/primarily (III-a)	2077/16,543	12.6	30/314	9.6
Street drugs minus cannabis primarily (III-b)	6065/16,543	36.7	62/314	19.8
Mono-substance use	10,427/16,543	63.0	254/314	80.9
Recent use of primary/only substance				
No use in last 30 days	2193/15,576	14.1	12/258	4.7
≤ 1 day a week	923/15,576	5.9	8/258	3.1
2–3 days a week	1386/15,576	8.9	16/258	6.2
4–6 days/week	1775/15,576	11.4	22/258	8.5
Daily	9299/15,576	59.7	200/258	77.5
Type of treatment				
Outpatient treatment	5254/16,543	31.8		
Inpatient treatment:				
Inpatient, non-hospital	2064/16,543	12.5		
Psychiatric hospital	3897/16,543	23.6		
General hospital (psychiatric service)	5220/16,543	31.6		
Treatment for criminal law offenders	108/16,543	0.7		
Source of referral				
GP	4515/16,543	27.3		
Care services for substance use problems	2349/16,543	14.2		
Hospital	5277/16,543	31.9		
Medical-psycho-social services	4402/16,543	26.6		

We summarized the type of substance use into mutually exclusive groups to fit the observed use across settings. Group I covers the use of alcohol only. Group II spans the use of pharmaceuticals, i.e. hypnotics, sedatives or pharmaceutical opioids, i.e. mainly opioid analgesics. Group III encompasses the use of street drugs, i.e. opiates, cocaine, stimulants other than cocaine, cannabis, hallucinogens and volatile inhalants. Group III was divided into two groups with group III-a spanning the use of cannabis only or primarily. Group III-b contains any other use of street drugs but no primary cannabis use and is further described as ‘street drugs minus cannabis primarily’. The classification of the three groups is hierarchical in the sense that the use of pharmaceuticals (group II) may be combined with alcohol (group I), while the use of street drugs (group III) may be combined with alcohol (group I) and pharmaceuticals (group II). Methadone, buprenorphine and fentanyl were classified in group III.

### Analysis

All data are episode-based. We used 95% proportion confidence intervals (CI) to describe patient population characteristics and bivariate associations. We used stepwise backward multiple logistic regression analysis to examine the research questions. Patient population characteristics that were significantly (*p* < 0.05) associated with the dependent variables were included in the full models. We accounted for clustering of patients within practices or treatment centres by using robust standard errors. Interaction effects between independent variables were tested only in the multivariable logistic model examining the second research question. Data were analysed with Stata 15.

## Results

### Sample description

The TDI register covered 60,310 episodes and the SGP network reported 314 episodes. A sample of 48,312 TDI episodes with a national identification number (NIN) showed that 28.7% of patients had more than one treatment period. No NIN are used by the SGP but proxy-indicators (age, sex, …) revealed 11 patients (3.6%) with more than one treatment period.For the first research question, we excluded TDI episodes from self-referred patients (*n* = 26,950; 46.0%), motivated by peers (*n* = 8881; 15.2%) or by court, probation or police (*n* = 6152; 10.5%). Excluding 33 episodes with invalid substance use data, we thus included 16,543 of 60,310 (27.4%) TDI episodes concerning patients that were referred by caregivers. Four types of referring caregivers were distinguished: GPs (*n* = 4515, 27.3%), care services for substance use problems (*n* = 2349, 14.2%), general or psychiatric hospitals (*n* = 5277, 31.9%) and (other) medical-psycho-social services (*n* = 4402, 26;6%).

Table 2 describes the characteristics of 16,543 treatment episodes reported to the TDI and the characteristics of 314 treatment episodes reported by the SGP network. The median age of the first use of the primary or only problem substance among TDI patients was 17 (Interquartile range (IQR): 15–21) and 18 (IQR 16–25) among SGP patients. In both populations most patients had alcohol problems only. Compared to the SGP population, the use of street drugs was higher in the TDI population, especially street drugs minus cannabis primarily (group III-b), while the use of pharmaceuticals (and alcohol) (group II) was lower. More than half (53.5%) of hypnotics and sedatives in the TDI population was used together with street drugs and thus classified in group III. In the SGP population pharmaceuticals were mostly combined with alcohol (70.8%) and thus classified in group II.

### How do patients starting specialist treatment for substance use problems on referral by their GP compare to similar patients referred by non-GP caregivers?

All socio-demographic and substance use characteristics of GP-referred patients and patients referred by other caregivers were significantly different (Table 3). GP-referral was also associated with type of treatment: almost half of the GP-referred patients started general hospital-based treatment, while GP-referral to inpatient treatment outside the hospital setting was rare. In the Walloon region, relatively more patients were referred by their GP and relatively fewer in the Brussels region.

**Table 3:** New episodes of specialist treatment for substance use problems by source of referral, TDI surveillance, Belgium 2016–7 (*N* = 16,543)

	Non-GP caregiver *n* = 12,028		GP *n* = 4515		Adjusted OR (95% CI) for referral by GP (versus non-GP caregiver) (*n* = 12,032)
	n/N	%(95% CI)	n/N	%(95% CI)	
Sex: man	8489/12,013	**70.7 (69.8–71.5)**	2987/4510	**66.2 (64.8–67.6)**	Removed ^b^
Age ≥ median	5659/11,999	**47.2 (46.3–48.1)**	2668/4507	**59.2 (57.7–60.6)**	1.25 (1.13–1.38)
Secondary educational level or higher	6840/9896	**69,1 (68.2–70.0)**	3172/3981	**79,7 (78.4–80.9)**	1.27 (1.15–1.41)
Recently employed	2000/10,788	**18.5 (17.8–19.3)**	1394/4201	**33.2 (31.8–34.6)**	1.71 (1.56–1.88)
Recent stable accommodation	8846/11,736	**75.4 (74.6–76.2)**	4229/4483	**94.3 (93.6–95.0)**	3.62 (3.08–4.26)
First treatment	3183/11,654	**27.3 (26.5–28.1)**	1904/4385	**43.4 (41.9–44.9)**	1.72 (1.57–1.87)
Type of substance use					
Street drugs minus cannabis primarily (III-b)	4748/12,028	**39.5 (38.5–40.3)**	1174/4515	**26.0 (24.7–27.3)**	reference
Alcohol only (I)	4827/12,028	**40.1 (39.3–41.0)**	2527/4515	**56.0 (54.5–57.4)**	1.10 (0.94–1.29)
Pharmaceuticals (and alcohol) (II)	799/12,028	**6.6 (6.2–7.1)**	391/4515	**8.7 (7.9–9.5)**	1.24 (1.04–1.48)
Cannabis only/primarily (III-a)	1654/12,028	**13.8 (13.1–14.4)**	423/4515	**9.4 (8.5–10.3)**	0.73 (0.62–0.86)
Mono substance use	7155/12,028	**59.5 (58.6–60.4)**	3272/4515	**72.5 (71.1–73.8)**	1.23 (1.04–1.48)
First use of primary/only substance at ≥17 years	4668/8475	**55.1 (54.0–56.1)**	1896/3067	**61.8 (60.1–63.5)**	^a^
Recent daily use of primary/only substance	6372/11,308	**56.3 (55.4–57.3)**	2927/4268	**68.6 (67.2–70.0)**	1.46 (1.33–1.59)
Type of treatment					^a^
Outpatient treatment	4022/11,920	**33.7 (32.9–34.6)**	1232/4515	**27.3 (26.0–28.6)**	
Inpatient, non-hospital	1928/11,920	**16.2 (15.5–16.8)**	136/4515	**3.0 (2.5–3.6)**	
Psychiatric hospital	2914/11,920	**24.4 (23.7–25.2)**	983/4515	**21.8 (20.6–23.0)**	
General hospital/psychiatry	3056/11,920	**25.6 (24.9–26.4)**	2164/4515	**47.9 (46.5–49.4)**	
Region of SGP					
Flemish	7180/12,028	59.7 (58.8–60.6)	2756/4515	61.0 (59.6–62.5)	Removed ^b^
Walloon	3133/12,028	**26.0 (25.3–26.8)**	1362/4515	**30.2 (28.8–31.5)**	
Brussels	1715/12,028	**14.3 (13.6–14.9)**	397/4515	**8.8 (8.0–9.7)**	

Non-overlapping confidence intervals are in bold

^a^ First use of primary substance was not included in the multivariable logistic model because of the low number of valid data and its high association with the type of substance use. Type of treatment was not included in the multivariable logistic model as it is not a socio-demographic patient characteristic or a substance use characteristic

^b^ Variable was removed because it did not significantly improved the fit of the model

Socio-demographic determinants of being referred by a GP were: higher age, higher education, recent employment and stable accommodation. Substance use determinants of a GP-referral were: first treatment episode, recent primary substance use and mono-substance use. Using street drugs minus cannabis primarily (III-b) as a reference category, GP-referred patients had (borderline) higher odds for using alcohol only (I) or pharmaceuticals (and alcohol) (II) and lower odds for using cannabis only/primarily (III-a).

### How do patients starting GP treatment for substance use problems without receiving concurrent specialist treatment compare to those who did?

For 9 out of 314 patients it was unknown whether they were receiving specialist treatment at the start of a new GP treatment episode, while 27.9% (85 of 305) patients did so. For 13 out of 85 patients the type of treatment was not reported, 53 patients received outpatient treatment and 19 inpatient treatment.

Table 4 shows significantly different socio-demographic and substance use characteristics between patients starting GP treatment only and patients who were concurrently receiving specialist treatment. Determinants of receiving GP treatment without concurrent specialist treatment were no previous treatment episode, being at work and living in the Walloon or Brussels region.

**Table 4:** New episodes of GP treatment for substance use problems without and with concurrent specialist treatment, SGP surveillance, Belgium 2016–7 (*N* = 305)^a^

	GP treatment only (no concurrent treatment) *n* = 220		Concurrent specialist treatment *n* = 85		Adjusted OR (95% CI) for GP treatment only (*n* = 259)
	n/N	% (95% CI)	n/N	% (95% CI)	
Recently employed	113/207	**54.6 (47.5–61.5)**	18/76	**23.7 (14.7–34.8)**	2.58 (1.36–4.91)
First treatment	91/198	**46.0 (38.9–53.2)**	13/79	**16.5 (9.1–26.5)**	2.78 (1.39–5.55)
Type of substance use					
Street drugs minus cannabis primarily (III-b)	37/220	16.8 (12.1–22.4)	23/85	27.1 (18.0–37.8)	Removed ^c^
Mono substance use	188/220	**85.5 (80.1–89.8)**	60/85	**70.6 (59.7–80.0)**	Removed ^c^
Recent use of primary substance	187/191	97.9 (94.7–99.4)	54/61	88.5 (77.8–95.3)	Not included ^b^
Region					
Flemish	126/220	**57.3 (50.4–63.9)**	64/85	**75.3 (64.7–84.0)**	Reference
Walloon	65/220	29.5 (23.6–36.0)	16/85	18.8 (11.2–28.8)	1.97 (1.06–3.66)
Brussels	29/220	**13.2 (9.0–18.4)**	5/85	**5.9 (1.9–1.3)**	

Sex, age and age of first use of primary substance were not significantly associated at the univariate level with receiving concurrent specialist treatment or not

Non-overlapping confidence intervals are in bold

^a^ For 9 of 314 patients it was unknown whether they were receiving concurrent specialist treatment

^b^ Recent use of primary substance was not included in the initial multivariate logistic model due to the small number of positive cases

^c^ Variable was removed because it did not significantly improved the fit of the model

## Discussion

Our main findings are twofold. First, the TDI data show that patients starting specialist treatment for substance use problems on referral by their GP have a distinct, more favourable profile compared to patients who were referred by non-GP caregivers. They were relatively older and socially better-off considering their education, employment status and their stable housing status. They were also better off regarding substance use problems with relatively more problems of alcohol and/or pharmaceuticals, more mono-substance use and first treatment episodes. There is some evidence that problem use of alcohol alone is less severe than poly substance use or street drug use [9, 10]. Second, the SGP showed that patients starting GP treatment without receiving specialist treatment were also better off compared to similar patients receiving concurrent specialist treatment. Among the latter, fewer patients were recently employed and more had been in treatment before. We thus found evidence confirming our assumption that patients starting GP treatment and concurrently receive specialist treatment are more similar to GP-referred patients starting specialist treatment compared to patients starting GP treatment only.

This study found considerable agreement between two data sources about general practice patients with substance use problems. New knowledge was acquired about the (referred) general practice population, such as education and recent accommodation. Yet, nearly 13% of educational data were missing in the TDI.

Our study has other weaknesses. One limitation is that data were compared on an aggregated level. So far, it has been impossible to measure overlap between the two surveillance systems at the patient level by unique patient identifiers. Due to the cross-sectional design of both the studies this paper presents a mere snapshot of the populations at a given point in time. Consequently, we cannot tell whether patients started GP treatment before and/or after starting specialist treatment. Neither do we know whether patients starting specialist treatment also receive(d) GP treatment.

Yet, the essence of a treatment episode clearly differs across levels of care. As described above (see “Sample description”), the percentage of patients with more than one treatment episode was much higher in the TDI (28.7%) than in the SGP (3.5%), despite uniform definitions. Several reasons may account for this large difference. In the TDI, a treatment trajectory may include subsequent treatment episodes in different settings, e.g. hospital-based detoxification first, followed by drug-free therapy in another setting. In general practice the difference between a health problem episode and a treatment episode is relatively small, especially as to unhealthy lifestyle. Moreover, the difference between new and ongoing problems and treatment is equally blurry, especially when chronic problems are concerned. In contrast to specialist treatment, substance use may not be an issue in every GP-patient contact during a treatment episode, even when the problem is still present. Conversely, the end/start of a treatment episode is much clearer in specialist treatment: when the patient fails to show up/shows up again. Given those limitations, it is impossible to estimate the size of the gap in the TDI register due to its non-coverage of general practice.

A PubMed search (September 2019) using the medical subheadings of ‘substance-related disorders/epidemiology’ and ‘general practice’ did not reveal recent papers with comparable research questions. Papers with (comparable) findings from the EMCDDA TDI were not found in PubMed. The focus of the SGP pilot study was different but some core results are comparable, e.g. type of substance use, previous treatment episodes and regional differences [5]. The pilot showed that 7 months after the baseline recording of new and ongoing episodes of GP-treatment, 21% of the patients who continued GP treatment also received specialist treatment. This proportion is similar to the proportion of 28% episodes of concurrent specialist treatment we found in this study.Monitoring treatment demand in general practice is one way of dealing with the problem of underdiagnosis of substance use problems, mostly alcohol, in general practice [11]. Yet, ‘detected’ patients in general practice may have more severe problems resulting in a relatively higher rate of referrals and concurrent specialist treatment. Unfortunately, we did not find evidence to verify this assumption.

Our findings fit the knowledge about general practice and GP care of patients with chronic, recurrent substance use problems. According to a good health services model, GPs provide as much care as possible to patients and refer patients to specialist health facilities only when more complex care is needed [12]. Continuity is a major attribute of general practice care, comprising continuing care over a lifetime, across health conditions and levels of care. In the context of substance use problems, this means preventive care, e.g. active screening and short interventions, and, aftercare or chronic patient care, e.g. patient support in case of relapse [13]. The finding that relatively more GP-referred and GP-treated patients were using the primary substance daily in the last 30 days may be exemplary of the chronic care role of GPs towards patients with, most likely, problems of alcohol and/or pharmaceuticals. Maybe those patients seek help from their GP in times of crisis: when they are drinking too much or have relapsed into drinking. GPs may be less strict than specialist caregivers about abstinence as a condition for starting treatment, but the widespread availability of alcohol and, to a lesser degree, pharmaceuticals, possibly also play a role.

This study yielded useful information for health policy and research. We found that GPs meet the demand of a specific population with substance use problems. This population is better off in more than one way. They may prefer to seek discreet help from their GPs above having to interrupt their social/work activities and seek specialist treatment, often outside the community. In this study, a relatively small part of the TDI population was examined. We believe that further research of motivators and referring caregivers of patients starting specialist treatment for substance problems would be useful to profile the population and treatment demand.

## Conclusions

We found considerable agreement between the SGP surveillance and the TDI-surveillance about the general practice population starting a new treatment episode for substance use problems in Belgium over a 2-year period. Examining the two data sources yielded new knowledge about the general practice population that is treated for substance use problems, more specifically, its relatively favourable profile.

## Data Availability

The datasets generated and/or analysed during the current study are not publicly available due to lack of resources but they are available from the corresponding author on reasonable request.

## Abbreviations

CI: Confidence interval
GP: General practitioner
OR: Odds ratio
SGP: Sentinel General Practices
TDI: Treatment Demand Indicator
